# Tryptophan usage by *Helicobacter pylori* differs among strains

**DOI:** 10.1038/s41598-018-37263-6

**Published:** 2019-01-29

**Authors:** Diana F. Rojas-Rengifo, Cindy P. Ulloa-Guerrero, Markus Joppich, Rainer Haas, Maria del Pilar Delgado, Carlos Jaramillo, Luisa F. Jiménez-Soto

**Affiliations:** 1 Molecular Diagnostic and Bioinformatics Laboratory, Biological Sciences Department, Los Andes University, Carrera 1 Nr.18A-10, Bogotá, Colombia; 2 Max von Pettenkofer Institute of Hygiene and Medical Microbiology, Faculty of Medicine, LMU Munich, Pettenkoferstr. 9a, D-80336 Munich, Germany; 3 Lehr- und Forschungseinheit Bioinformatik. Institut für Informatik, Ludwig-Maximilians-Universität München, Amalienstr. 17, D-80333 Munich, Germany; 4 Ludwig-Maximillians University, Munich, Germany

## Abstract

Because of its association with severe gastric pathologies, including gastric cancer, *Helicobacter pylori* has been subject of research for more than 30 years. Its capacity to adapt and survive in the human stomach can be attributed to its genetic flexibility. Its natural competence and its capacity to turn genes on and off allows *H. pylori* to adapt rapidly to the changing conditions of its host. Because of its genetic variability, it is difficult to establish the uniqueness of each strain obtained from a human host. The methods considered to-date to deliver the best result for differentiation of strains are Rapid Amplification of Polymorphic DNA (RAPD), Multilocus Sequence Typing (MLST) and Whole Genome Sequencing (WGS) analysis. While RAPD analysis is cost-effective, it requires a stable genome for its reliability. MLST and WGS are optimal for strain identification, however, they require analysis of data at the bioinformatics level. Using the StainFree method, which modifies tryptophan residues on proteins using 2, 2, 2, - trichloroethanol (TCE), we observed a strain specific pattern of tryptophan in 1D acrylamide gels. In order to establish the effectiveness of tryptophan fingerprinting for strain identification, we compared the graphic analysis of tryptophan-labelled bands in the gel images with MLST results. Based on this, we find that tryptophan banding patterns can be used as an alternative method for the differentiation of *H. pylori* strains. Furthermore, investigating the origin for these differences, we found that *H. pylori* strains alters the number and/or position of tryptophan present in several proteins at the genetic code level, with most exchanges taking place in membrane- and cation-binding proteins, which could be part of a novel response of *H. pylori* to host adaptation.

## Introduction

*Helicobacter pylori* is a Gram (−) bacterium colonizing the human stomach. It has been estimated that around 50% of the human population are colonized by *H. pylori*, most of them during childhood^1,2^. Several studies have confirmed the evolution of *H. pylori* in the host^3–5^, and its genetic changes in relation with time has been attributed to its capacity to incorporate external genetic material into their DNA. For research studies as well as for diagnosis, the identity of a bacterial strain relies on its DNA stability. Based on this premise, one of the preferred methods for comparison of strains and their identity is the RAPD (Rapid Amplification of Polymorphic DNA). This technique uses a short DNA primer containing a sequence that is able to anneal to several regions of the DNA amplifying fragments variable in size and creating a pattern of fragments after their separation in a gel. This method is relatively easy to establish in a molecular analysis laboratory, assuming that PCR is part of the routine work. However, since RAPD is based on DNA stability between strains, *H. pylori* poses here a challenge. The capacity of *H. pylor*i to acquire and randomly incorporate DNA into its own genome (natural transformation competence) makes RAPD test results somehow unreliable for this bacterium. At the moment, there are two methods recognized to distinguish *H. pylori* strains from each other: Multi-locus Sequence Typing (MLST), which relies in the stability of seven highly conserved genes in the genome of *H. pylori*, and Whole Genome Sequencing (WGS). For MLST fragments ranging from 400 to 600 bp each of seven genes (*atpA*, *efp*, *mutY*, *ppa*, *trpC*, *ureI* and *yph)*, are amplified and their products are sequenced. The results are then used to confirm the uniqueness of the isolates^6^. This method, although highly accurate, implies higher costs than the detection of DNA fragments and their patterns as in RAPD. In the case of WGS, even though the advancement of sequencing technologies has reduced the costs for whole genome sequencing to hundreds of dollars per genome, the results require extensive and specialized bioinformatic analysis, not always available or suitable for all purposes. Tryptophan is the biggest amino acid existing, and with methionine, they are the only two amino acids encoded by only one codon (UGG and AUG, respectively). Tryptophan is considered an essential amino acid and its indole group makes it an aromatic amino acid with paramagnetic characteristics^7^ and confers proteins a fluorescence at 280 nm wavelength^8^. Ladner *et al*. adapted the use of 2, 2, 2 trichloroethanol (TCE) to stain proteins in polyacrylamide gels making all proteins containing tryptophan residues visible^9^, by adding chloride to the indole ring, which changes the excitation wavelength of tryptophan to 305 nm with an emission at around 500 nm. When visualizing full protein lysates from *H. pylori* strains with this method, each strain presents a distinguishable pattern of proteins containing tryptophan. The analysis of each strain’s pattern (or tryptophan fingerprint, TryF) was compared with MLST data, showing TCE staining of proteins (StainFree method) as a reliable alternative method for the differentiation of *H. pylori* strains, with lower costs and faster results than the currently available ones.

Protein separation in 1D acrylamide gel is governed by different protein characteristics, like their size, posttranslational modifications and conformation^10–16^. The detection of proteins using tryptophan, is not only dependent on the number amount of tryptophan residues present in a protein’s primary structure, but as well on the protein expression levels. Tryptophan is usually encoded by only one codon (UGG). However, yeast mitochondria and *Mycoplasma capricolum* have adapted the stop codon UGA to code for tryptophan instead^17–19^. In *H. pylori* there appears to be no biased codon usage^20^ and exchange of stop codon for tryptophan has not yet been described.

Here we validated the use of tryptophan patterns from full protein lysates of *H. pylori* as a method for identification of strain uniqueness and investigated if different usage changes of tryptophan in *H. pylori* proteins by each strain are encoded in the bacterial genome and which implications this might have.

## Results

### Tryptophan Fingerprinting detects variation within *H. pylori* strains undetected in RAPD

**Figure 1:**
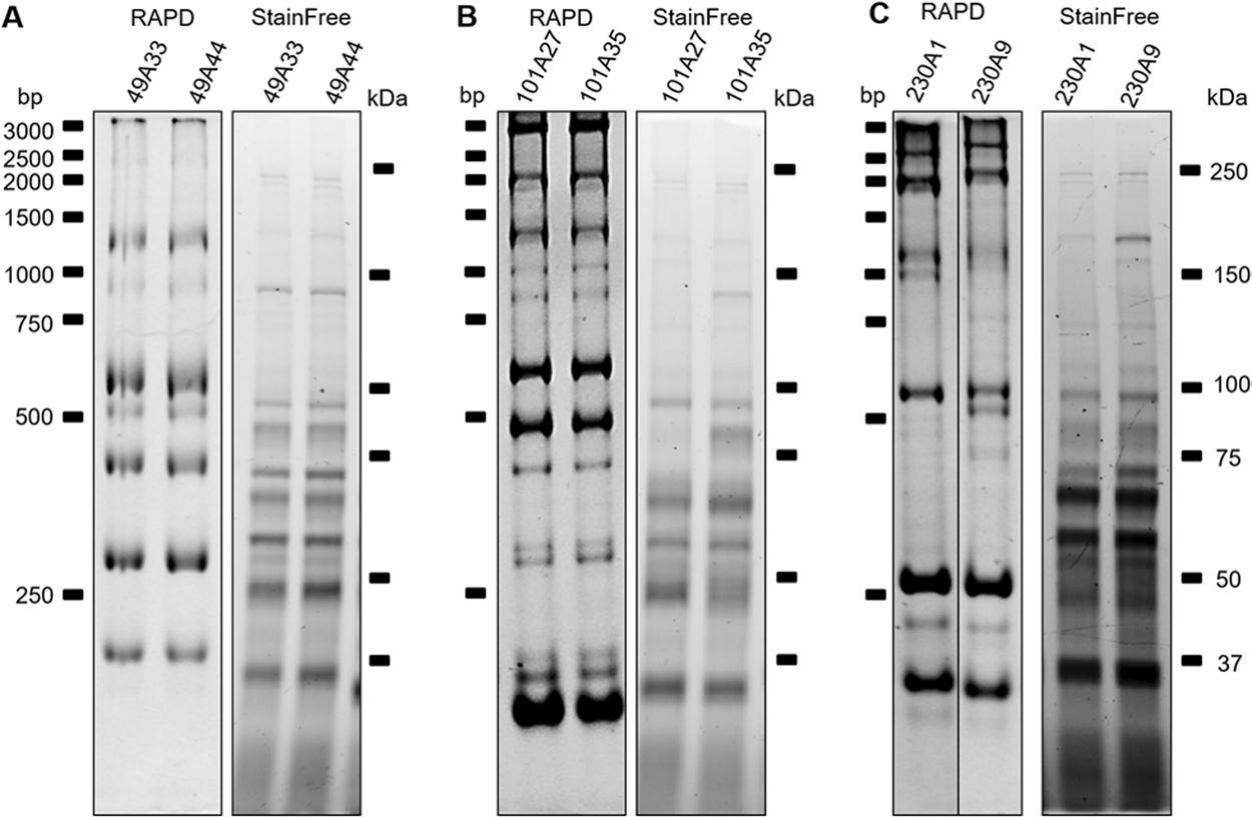
RAPD and Tryptophan Fingerprinting (TryF). Two *H. pylori* strains isolated from patients 49 (**A**), 101 (**B**) and 230 (**C**) were analyzed using RAPD (DNA analysis) and StainFree (Tryptophan in proteins). For comparison of signals, original images were cropped and fitted for better graphic display. Original images and their metadata are available in supplements (Supplementary images).

Random Amplification of Polymorphic DNA (RAPD) is a fast and efficient way to evaluate the genomic identity of two strains, and it is used routinely in laboratories to confirm the uniqueness and/or identity of a strain. We had seen differences in tryptophan patterns for standard strains previously^21^ and here decided to compare RAPD and tryptophan patterns from six *H. pylori* strains isolated from three patients (two per patient). In different strain pairs, different results can be observed. For example, RAPD results for strains from patient 49 show both strains as being identical, which is confirmed with the presence of identical tryptophan pattern (Tryptophan Fingerprinting (TryF) for the same strains) (Fig. 1A). However, strains with identical RAPD can show differences in TryF, as it was seen with strains from patient 101 (Fig. 1B). As expected, differences between strains were as well established by both methods (RAPD and TryF) as it was the case for strains from patient 230 (Fig. 1C). Cases for which RAPD showed differences between strains and TryF showed them to be identical were not observed in any of the 43 patient’s strains analyzed.

### TryF (Tryptophan Fingerprinting) method for strain identity is as reliable as MLST in *H. pylori* strains

**Figure 2:**
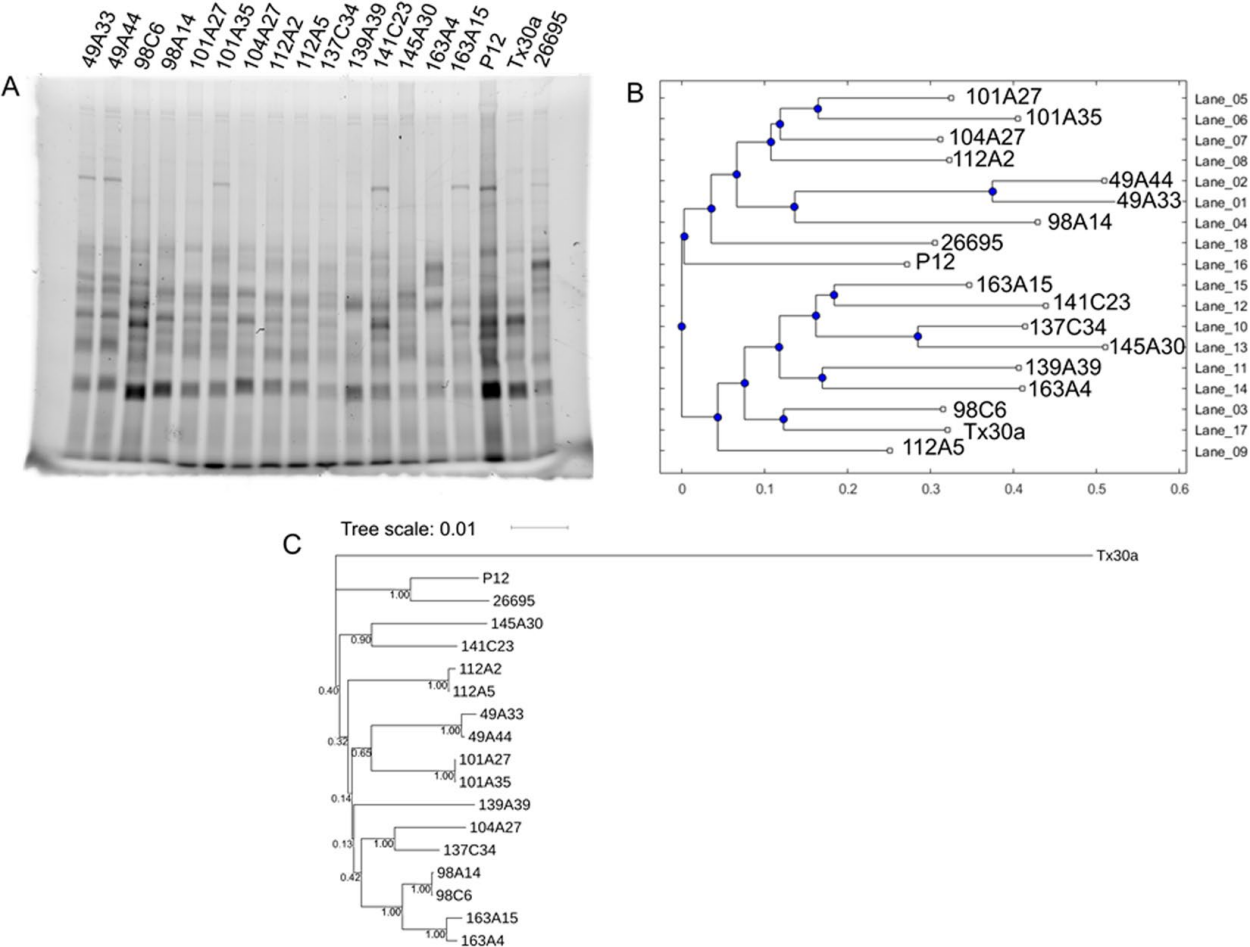
Data analysis from Tryptophan Fingerprinting (TryF) and MLST. (**A**) Protein samples from *H. pylori* full lysates in single-gel 1D acrylamide stained with TCE and detected at 500 nm after activation with 305 nm. The bands from this image were detected using 1D-GelPhyBase, and its analysis produced the dendrogram (**B**) showing the distances generated according to the dice similarity matrix between band pattern (Axis X) and lanes (Axis Y). (**C**) MLST analysis of same strains in gel and their distances. Numbers on roots show the support bootstrap values of the branches (0–1). Tree was constructed using a nucleotide substitution model of Kimura-2 parameters and a NJ clustering method with 10000 bootstraps.

Data comparison with RAPD and TryF showed that differences between strains could be visualized. Since MLST is considered more reliable for differentiation of strains in *H. pylori*, we decided to compare the TryF data with this method. For a better analysis and comparison with MLST data, we designed a tool for the detection of bands and its analysis using dendrograms to establish similarities between strains (Ruiz and Ulloa-Guerrero, *et al*., manuscript in preparation). Since any pattern comparison in 1D-gels can be only attempted if most external variables are reduced, all samples were standardized (see below) and all samples compared were loaded in the same gel. During detection of tryptophan in proteins, signals are dependent on the amount of protein contained in the sample and the number of tryptophan residues present in each protein. In our analysis, band presence and intensity were considered. Therefore, to avoid false detections, all protein lysates are standardized to equal amount of colony forming units (cfu) per sample (see Materials and Methods). Tryptophan was detected in the acrylamide gel after electrophoresis and analyzed using the 1D-GelPhyBase software (see Material and Methods for details). This software produced the corresponding dendrogram (Fig. 2B) showing similarities and dissimilarities in strains. As standard strains we used 26695, P12 and Tx30a. As expected, all three standard strains are recognized as independent strains with different relative distances between each other. Tx30a clustered separately from strains P12 and 26695. Even though P12 and 26695 belong to the same cluster, they are sister groups that have variations in the tryptophan pattern generating a slight difference in the relative distances of the branch lengths (Fig. 2B). When comparing the patient strains, only strains from patient 49 (49A44 and 49A33) seem to be closely related. However, strains from two different patients (139A39 and 163A4), which are expected to be independent strains, showed as well high similarity in this analysis. To confirm the uniqueness of all other strains, the MLST data from each strain was analyzed (Fig. 2C) and showed that strains from patient 112 (112 A2 and 112A5), 49(49A33 and 49A4), 101 (101A27, 101A35), 98 (98A14 and 98C6) and 163 (163 A15 and 163A4) are the most similar, while all other ones are different enough to be potentially unique strains. To clarify the identity of the patient strains, we performed genotyping using *cagA*, *vacA* and *hopQ* genes and immunological tests for phenotyping by identifying the production of CagA, VacA and OipA. For *cagA* gene we amplified the region encoding the EPIYA motif at the 3′-half of the gene. Although *vacA* is present in all strains, it differs between strains in two regions: region s (5′-region) and region m (middle region)^22^. *hopQ* gene is known to have two distinct alleles (Allele I and Allele II) that show some correlation with *cagA* gene^23^. The existence of *cagA* and *vacA* genes are better evaluated through detection of protein production. Therefore, this data has been complemented with immunodetection of CagA and VacA. In the case of OipA, an Outer Membrane Protein, it is known to be turned on and off by *H. pylori* through slipped-strand mispairing^24^. Its relevance in infection requires detection of the protein for a correct typing of the strain. With genotype and phenotype data (Supplementary Table 1) the differences in strain classification between TryF and MLST are clear: In case of the MLST analysis, only strains from patients 49 and 112 show identical phenotypes and genotypes in the genes tested. However, in the case of patient 101, one of the strains contains the gene for CagA but it does not express it; for patient 98, only strain 98A14 expresses enough VacA that was detectable in our assay; and for patient 163 both strains are only similar in the allele type of *hopQ* (allele II). These differences between strains also explain the differences between the dendrograms of TryF and MLST. While the MLST dendrogram graphs the phylogenetic distance of single strains (with Tx30a being the most distant), the TryF method detects differences in protein production, which creates a different kind of distance measure, more related to phyloproteomics.

In order to calculate the accuracy of this method a Gold Standard for declaration of uniqueness of a strain, was defined as the combination of MLST results and genotyping of strains. Based on the three methods used: RAPD, TryF and MLST, 44 strains were classified as “unique” or “not unique” (See Supplementary Table 2) and their results summarized in Supplementary Table 3. The Cohen’s Kappa Coefficient was calculated (see Table 1) resulting in TryF with a kappa (k) of 0.72 (Sensitivity 0.94, Specificity 0.82) in contrast to MLST alone (k = 0.37, Sensitivity 0.5, Specificity 0.86).

**Table 1:** Cohen Kappa Coefficients from comparison of golden standard (MLST complemented with genotyping) with TryF, RAPD and MLST. With PVV: Positive predictive value, and NPV: Negative predictive value.

	Cohen’s Kappa (*k*)	Sensitivity	Specificity	PPV	NPV
***Gold Standard***					
*TryF*	0.72	0.94	0.82	0.75	0.96
*RAPD*	0.23	0.69	0.57	0.48	0.76
*MLST*	0.37	0.50	0.86	0.67	0.75

Finally, these results lead us to conclude that TryF is as reliable as, or even better, method than MLST to detect the uniqueness of strains since its interpretation considers protein production.

### Differences in Tryptophan usage by *H. pylori* strains is genetically encoded

**Figure 3:**
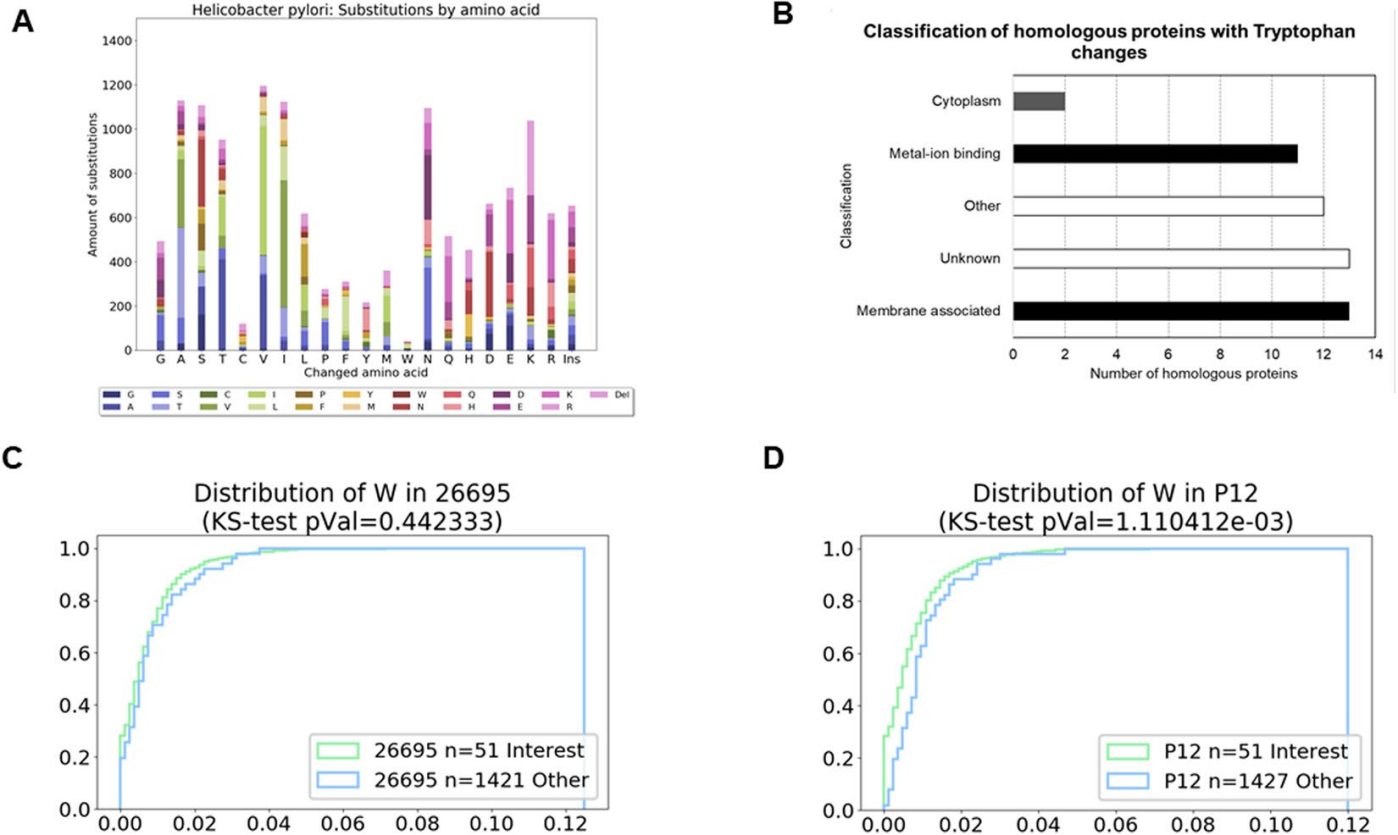
Comparisons between homologous proteins in *H. pylori* strains 26695 and P12 at their amino acid level. (**A**) Amino acid exchanges, deletions (Del) and insertions (Ins) between all homologous proteins. X axis show amino acid residues present in 26695 proteins and insertion events. Bars show the type of amino acid exchanges occurred in P12 (See legend for color code of amino acids). (**B**) Classification of the 51 homologous proteins showing a higher than expected exchanges of tryptophan residues using as source the Gene Ontology, Pfam and InterPro information. X axis shows the numbers or residues exchanged for tryptophan, while Y shows the classes for the homologous proteins. (**C**,**D**) Cumulative histograms for tryptophan content in the 51 proteins compared to the rest of proteome. Results of the Kolmogorov-Smirnov test (KS) for each comparison are shown under the title.

Direct change in the genetic code to include or replace an amino acid is the most efficient way to change protein compositions, and therefore an efficient way to change the amount of tryptophan present in proteins. To define the relevance of genomic changes in tryptophan composition between different strains in *H. pylori*, two reference strains (P12 and 26695) were used to compare the changes on Tryptophan (Trp, W) in their homologous proteins. These strains showed differences experimentally in their tryptophan banding pattern of full lysates experimentally analyzed on gels (Fig. 2A). The analysis of their genomes showed that 106 homologous proteins have changes in tryptophan content by deletion, insertion or exchange of amino acids, counting to a total of 99 tryptophan residues changed. Comparing to other amino acids exchanges (Fig. 3A), tryptophan has the lowest exchange rate, followed by cysteine and tyrosine. Looking in detail the tryptophan exchanges between P12 and 26695, most of the changes occurred by exchanges between tryptophan and leucine (W/L, 41 cases (44%)), followed by glycine (W/G, 8 cases (8%)), deletions (6 (6%) and insertions (5 (5%)). From all tryptophan residues in the homologous proteins (P12(CP001217): 2720, 26695 (AE000511): 2713), 2672 remain constant. There were no exchanges detected between the ring-containing amino acids (Proline (P), Histidine (H)); methionine (M) and glutamic acid (E) when comparing these two strains. Exchanges with other aromatic amino acids were present (Phenylalanine (4%), Tyrosine (3%)). However exchanges with less favorable amino acids, like leucine and/or glycine, superseded the favorable exchanges with other aromatic amino acids^25^.

### Several tryptophan exchanges could be correlated with the function of proteins

Changes of amino acids in proteins are expected in any organism, therefore we filtered the data to select only those homologous proteins where the changes in tryptophan were higher than expected by the general mutation rate (see computational analysis in Material and Methods). This classification resulted in 51 proteins with higher than expected tryptophan changes and were classified based on the Gene Ontology (UniProt), Pfam and IntelProt information available for each (Supplementary Table 6). Based on this information they were classified into the general classes Membrane (Integral Component of the Membrane (GO:0016021), Membrane Transport (GO:0055085), Plasma Membrane(GO:0005886) Membrane (GO:0016020) and Outer Membrane Proteins)), Cation binding ((Metal binding GO:0046872), Zinc Ion binding (GO:0008270), Cobalt-binding domain), Cytoplasm (GO:0005737), DNA binding (GO:0003677) and others.

As shown in Fig. 3B, most of the homologous proteins with changes in their tryptophan content belong to the Membrane group (25%), followed by Cation binding proteins (20%). From the 51 proteins, 14 (27%) could not be classified in any of the groups.

Figure 3C,D are cumulative histograms for P12 and 26695 strains, highlighting the total amount of tryptophan genetically encoded in all proteins compared to the 51 homologous proteins discovered previously. It shows that although both strains have shown a similar tryptophan content in general, the 51 homologous proteins in P12 have a higher amount of tryptophan in relation to the rest of the proteome (P < 0, 001), while in 26695 this difference is not significant (P = 0, 44), highlighting the targeted tryptophan changes specific to each strain.

Tryptophan can alter the interaction of proteins with membranes^26,27^. Since most of the proteins with tryptophan exchanges were membrane associated proteins, we inspected more closely the tryptophan exchanges across all homologous proteins in *H. pylori*. Using the 26695 locus tag for the different proteins, we performed a BLAST search in *Helicobacter pylori* (Taxid:210) using a BLOSUM80 matrix. The best 250 hits from these results were then aligned using Cobalt (NCBI), exported as a CLUSTAL alignment and visualized using MSAViewer^28^. The frequency of amino acids in the alignment for all homologous proteins with exchange of tryptophan are shown in Supplement Fig. S2. Analyzing the tryptophan exchanges in the membrane associated proteins (13), only three proteins had the exchange (W to (G/S/A/C)) in the transmembrane region, four proteins had deletion/insertions of peptides containing tryptophan, most of them N-terminal. All other exchanges including tryptophan and leucine where within a region highly hydrophobic, rich in leucine, valine and isoleucine residues.

### Tryptophan changes in *H. pylori* are more frequent than in the closely related species: *Campylobacter jejuni*

**Figure 4:**
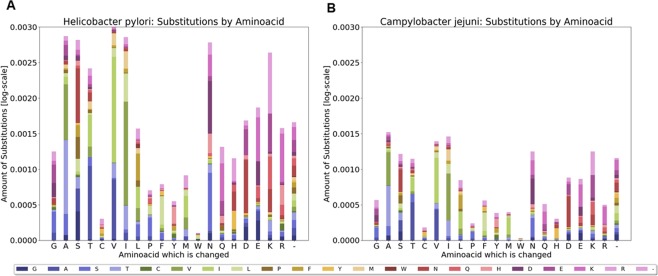
Comparison of relative amino acid exchanges in *H. pylori* and *C. jejuni* strains. For each bacteria species, the homologous proteins from two sequenced strains were compared and their amino acid exchanges are shown relative to their absolute number of amino acids present in their genome (Y axis). Amino acid residues changed are shown on X axis and the correspondent amino acid exchanges are shown in the bars. Color code shown on the legend.

To determine if variations in the tryptophan content is something particular to *H. pylori* strains, we compared the amino acid exchanges between two standard strains from *Campylobacter jejuni*, a closely related species to *H. pylori* and a member of the epsilon-proteobacteria. The number of exchanges was normalized to the number of amino acids residues present in each genome. Figure 4 shows that both *H. pylori* strains (P12 and 26695) have a higher exchange of amino acids than *C. jejuni* strains in almost all residues. In this context, *H. pylori* strains are approx. 3.5 times more likely to exchange the tryptophan in its proteins as compared to *C. jejuni* strains. Interesting is the observation that in some residues (G, A, S, T, D, E, K, and R) a similar proportion of exchanges can be observed, while in others (W, C, P, Y, M, N and H) higher variations in the amino acid exchanges between both species were found.

## Discussion

RAPD analysis for identification of unique DNA is not a reliable alternative for the analysis of *H. pylori* strains. Reliable methods like MLST and WGS come with higher cost and require time for its analysis. And even the interpretation of MLST data for the identification of identical strains is difficult with *H. pylori*. It has been documented that the high genetic variability of *H. pylori* strains in the host across time makes the certain genotyping of a strain challenging^29–32^. On the other hand, Tryptophan fingerprinting (TryF) was able to expose changes in bacteria that are only present at the protein level and not necessarily visible in the genomic analysis, allowing a quick and reliable identification of candidate identical strains. The TryF analysis should not be considered an absolute way to define the identity of a strain; however, it offers a quick and low-cost method to screen a high number of samples compared with the methods available and limit the amount of samples to sequence if necessary. While MLST and WGS can take up from one to several weeks for a final result, TryF can be performed in one day including the analysis with our developed software, making it the best method for first screening of strain identity. This is possible because of the differences that *H. pylori* presents in tryptophan usage in its strains.

To alter the number of tryptophan present in a protein, the bacterium must do any of the following: i) change its DNA to eliminate/add or alter a codon to match a UGG codon, ii) adapt or modify the Aminoacylsynthetase for the tRNA_Trp_, iii) modify its tRNA to recognize other codons, or iv) use the stop codon UGA as alternative codon for tryptophan (reviewed in Ling J *et al*.^33^). Additionally to genome changes, other causes for differences in tryptophan patterns observed in the protein gels include changes in proteins expression or their glycosylation. We opted to analyze the changes on genetic code and found that in the two reference strains (P12 and 26695) the changes observed in the protein gels can have a genetic background. Moreover, the changes here discovered showed that leucine codons (CCU, CUC, CUA, CUG, UUA, UUG) where preferential for changes to tryptophan (UGG). When compared to *Campylobacter jejuni* strains (a close related bacteria species from the epsilon-proteobacteria group), we found that *H. pylori* is three times more likely to exchange their tryptophan content between these strains. In general *H. pylori* has a higher variation of amino acid residues between homologous genes than the analyzed *C. jejuni* strain. This could be explained by the genetic flexibility of *H. pylori* as response to host interaction^34^.

Tryptophan (Trp, W) is one of two amino acids coded by only one codon. It is the biggest of the amino acids and its aromatic chain confers hydrophobic non-polar characteristics to the protein altering the protein’s hydrophobic mismatch response^35,36^. Additionally, as with all other aromatic amino acids (Tyrosine (Y), Phenylalanine (F)), tryptophan plays an important role in π-π and cation-π interactions involved in secondary, tertiary and quaternary conformation of proteins and their interactions with cations^37,38^. The fact that most of changes occurred in proteins with membrane interactions and cation-binding domains, support the hypothesis that tryptophan changes in *H. pylori* may play an important role in the adaptation of proteins to the needs of each strain. Since tryptophan is known to alter the association of proteins with membranes, it was a surprise to discover that most of the exchanges in membrane associated proteins did not take place in the transmembrane regions, but within regions rich in hydrophobic amino acids. Therefore, we presume that these changes might have a functional target. Several options have been described. Relevance for dimerization of the proteins was described for the TssL protein, part of the inner membrane components of the Type VI secretion system, through tryptophan π-π interactions^39^. Other possibilities include conformational switches with tryptophan residues, as it was shown in the gp120 of HIV^40^, or to alter the activity of the protein or its recycling through oxidation of tryptophan residues as cue^41^.

Although we do not yet know the specific cause for changes of tryptophan in proteins by *H. pylori*, we can use it to distinguish strains in a specific way and we open the possibility that this phenomenon could be used by other bacterial species. Further studies with other bacterial species would answer if these changes are part of a general physiological adaptation of bacteria to their environment or if it is a specific method by *H. pylori* to adapt to a changing host.

## Materials and Methods

### Bacterial culture

Bacteria were grown on solid agar plates (GC-agar, Oxoid) complemented with a vitamin mix and cholesterol/fatty acids mixture (Gibco, Thermo Scientific) as described previously^42^. All bacteria were grown between 18 to 24 hours in microaerophil conditions (5%O2; 10%CO2; 85%N2) at 37 °C and collected in PBS (Gibco, Thermo Scientific) for densitometry measurements in order to standardize all protein lysates.

### DNA extraction and protein sample preparation

Chromosomal DNA was extracted from *Helicobacter pylori* ‘s culture using a commercially available kit for Chromosomal DNA extraction from tissue and following manufacturer’s instructions (QIAGEN, Valencia, CA). For protein lysates, a suspension of 6 × 10^7^ bacteria (cfu, colony forming units) was centrifuged at 4000 rpm at 4 °C in a swing rotor centrifuge for 20 minutes. After removing the supernatant, the pellet was suspended in 20 μL cold PBS, mixed with 25 μL of 2X SDS loading buffer (non-reducing conditions) and boiled at 95 °C for 10 min for separation of protein using SDS-acrylamide single-gel system (see below). Samples, Chromosomal DNA and protein lysates were stored at −20 °C until necessary.

### RAPDs

Based on Akopyanz *et al*. protocol^43^, the primer used was: D9355 (2): CCGGATCCGTGATGCGGTGCG. A PCR final volume of 25 μL was carried out containing GoTaq 1× (Promega), 1 pmol of primer, water and 2 μL of DNA. PCR conditions were 5 cycles of (94 °C, 5 min; 40 °C, 5 min; and 72 °C, 5 min), 30 cycles of (94 °C, 1 min; 55 °C, 1 min; and 72 °C, 2 min), and then 72 °C, 10 min. After PCR, the products were electrophoresed on an 8% DNA polyacrylamide gel (30% Bis-Acrylamide (29:1), TAE 1×, 10% Ammonium persulfate, TEMED and water) for 1.5 hours at 25 mA constant. DNA in gel was visualized after ethidium bromide staining for 15 minutes and exposed to UV light on a ChemiDoc (Bio-Rad Laboratories, Inc., California, USA).

### StainFree staining

Single gel systems^44^ were adapted for StainFree detection as described in protocol depository Protocols.io under 10.17504/protocols.io.gipbudn.

### Multilocus Sequence Typing (MLST)

Corresponding DNA regions were amplified from *atpA*, *efp*, *mutY*, *ppa*, *trpC*, *ureI* and *yphC* genes, using the same protocol as described by Achtman *et al*.^45^. Each reaction was adjusted to a final volume of 25 μL containing GoTaq® Green Master Mix 2 × (Promega), 0.1 pmol/μL of each primer, 9.:7 μL of nuclease-free water and 2 μL of microbial DNA. The thermal profile has been previously described (Achtman *et al*.^45^) and was carried out in the C1000 Touch Thermal Cycler (Bio-Rad Laboratories, Inc., California, USA). The amplification products were separated on a 2% agarose gel in 0.5X TAE buffer (Tris/acetate/EDTA) at 70 V x 90 min and visualized with GelRed^TM^. The amplification products were purified and subsequently sequenced in Macrogen. Inc (Seoul, South Korea). Once obtained, the sequences were assembled with the CLC Genomics Workbench 8 software (CLC bio) and finally compared with reference sequences of *H. pylori* 26695 and J99 (access GenBank: NC 000915.1 and NC 000921.1, respectively). Based on the difference in their allelic frequency of these constitutive genes, phylogenetic analysis was performed using MEGA7 (Kumar, Stecher, & Tamura, 2016) applying Kimura’s two parameters model of nucleotide substitution and Neighbor-joining (NJ) clustering with a bootstrap of 10,000.

### Gel analysis

All gel images in greyscale were used to build a dendrogram using the 1D-GelPhyBase software (Ruiz and Ulloa-Guerrero, *et al*., manuscript in preparation). The first stage uses as input the raw gel electrophoresis image and translates it into a grey scale intensity matrix which can be used to perform the analysis. This program adjusts the image saturation and contrast and obtains the complementary image by implemented image processing functions. Subsequently, a rectangle is drawn on each lane for which bands are to be detected. For each rectangle, a band pattern image is generated and is converted from its image format a double type variable of intensity values. Changes in intensity along the lane were used to detect the location of bands. (Supplement Fig. S1). To remove most of this noise, a Savitsky-Golay Filter was implemented with a pre-existing function to smooth the intensity results^46^. The locations of the bands are defined as local maxima of the intensity profile using a threshold of 0.012 as the peak prominence to measure the change in intensity compared to neighboring regions. Data for each lane is stored in a matrix in which binary values are assigned; 1 for band presence, 0 for band absence. The result is a matrix profile of the location of bands in every lane. An admissible error parameter is defined to consider the magnitude of the distance that the homologous bands in different lanes could be apart. The matrix profile is then used to calculate DICE similarity coefficient and in turn generate a similarity matrix^47^. Finally, dendrograms are generated using similarity matrix with the Neighbor Joining (NJ) method. Scripts and documentation can be found at https://github.com/ha-ruiz75/BandDetection.git

### Genotyping strains and immunodetection of CagA, VacA and OipA

For the detection of the genes *cagA*, *vacA* (s and m regions) and *hopQ* alleles, the original protocols were followed for each of the genes (see Supplementary Table 4). For the immunodetection, the protocol followed for separation in gel was described above. For the transfer of proteins to a PVDF membrane the protocol is available in protocols.io under 10.17504/protocols.io.ghhbt36. The antibodies used have been previously described (see Supplementary Table 5). In summary, membranes were blocked using 3% BSA in PBS for one hour, primary antibody incubated for 1 hour at room temperature, washed 5 times 10 minutes each, secondary anti-rabbit antibody peroxidase conjugated (sigma) was used for detection after 45 minutes incubation followed by four wash steps 15 minutes each. Once detected, membranes were stripped for removal of antibodies and prepared for the following detection.

### Bioinformatic analysis

#### Homologous Proteins in H. pylori and C. jejuni

Using BLAST^48^, two proteomes of *Campylobacter jejuni* (Strain RM1221 (Genbank NC_003912), Strain NCTC 11168 (Genbank NC_002163)) and *Helicobacter pylori* (Strain P12 (ENA CP001217), Strain 26695 (ENA AE000511)) were aligned against each other for the identification of their homologous proteins (Joppich *et al*., Manuscript in preparation). Homology has been assumed if the sequence identity is higher than 0.9 and at least 80% of the larger protein is covered. This resulted in identification of 1543 homologous protein-pairs in *C. jejuni* and 1399 for *H. pylori*.

#### Amino acid replacement identification

A global alignment of the homologous proteins using the Blosum80 substitution matrix with gap costs of 50 and gap extension costs of 0.2 was performed, deriving specific substitution matrices for the two strains of *Helicobacter pylori* and *Campylobacter jejuni* (Fig. 4).

#### Identification of proteins with higher than expected tryptophan changes

For each of the homologous proteins, the relative amount of tryptophan was calculated. In general, the distribution of the relative amount of tryptophan per protein remains constant for both bacteria strains (data not shown).

For *H. pylori* 106 homologous proteins had tryptophan changes between strains. Since normal mutations of tryptophan can occur in a genome, we calculated the expected tryptophan exchange rate. This one was defined as the current amount of tryptophan residues in the protein minus the tryptophan losses plus the expected amount of tryptophan in the changed amino acids (relative tryptophan frequency in genome times number of changed amino acids).

With tryptophan being seldom changed, this probably overestimates the actual change in tryptophan. To consider a protein tryptophan exchange as higher than expected, the actual change (gain plus loss) must be higher than 0.2 if the expected count difference (expected value minus actual value in homologous protein) is larger than 1, or, if the actual change is less than 0.2, the expected count difference must be at least 0.8. This resulted in 51 proteins of high interest (See Supplementary Table 6).

#### Classification of proteins of interest

The identified 51 proteins with a higher-than-expected change in tryptophan were organized in classes based on their Gene Ontology^49^ classes of the corresponding genes shown in the Uniprot database (See Supplementary Table 6). For proteins lacking a GO number, a manual curation of the protein-genes was conducted using the Pfam and InterPro information available.

## Supplementary information

Supplementary Data

## Data Availability

This document’s data, protocols and methods have been made available in the supplementary section and in repositories. If additional data presented here is required, it will be made available upon request to the corresponding author.
