# Supplementary material for: Tryptophan usage by Helicobacter pylori differs among strains
Source: Sci Rep. 2019 Jan 29;9:873. doi: 10.1038/s41598-018-37263-6 (PMC6351589; doi:10.1038/s41598-018-37263-6)
Supplement: Supplementary file 1 — Supplementary Data [file 41598_2018_37263_MOESM1_ESM.pdf]

Supplementary Information

Tryptophan usage by *Helicobacter pylori* differs among strains

Diana F. Rojas-Rengifo[1,2], Cindy P. Ulloa-Guerrero[1], Markus Joppich[3],

Rainer Haas[2], Maria del Pilar Delgado[1], Carlos Jaramillo[1], and Luisa F. Jiménez-Soto[2,4]*

1. Molecular Diagnostic and Bioinformatics Laboratory, Biological Sciences Department, Los Andes University, Carrera 1 Nr.18A-10, Bogotá, Colombia.

2. Max von Pettenkofer Institute of Hygiene and Medical Microbiology, Faculty of Medicine, LMU Munich, Petternkoferstr. 9a, D-80336 Munich,Germany

3. Lehr- und Forschungseinheit Bioinformatik. Institut für Informatik. Ludwig-Maximilians-Universität München. Amalienstr. 17, D-80333 Munich, Germany

4. Ludwig-Maximillians University

* Corresponding author: L.Jimenez@campus.lmu.de.

Supplementary Figure S1

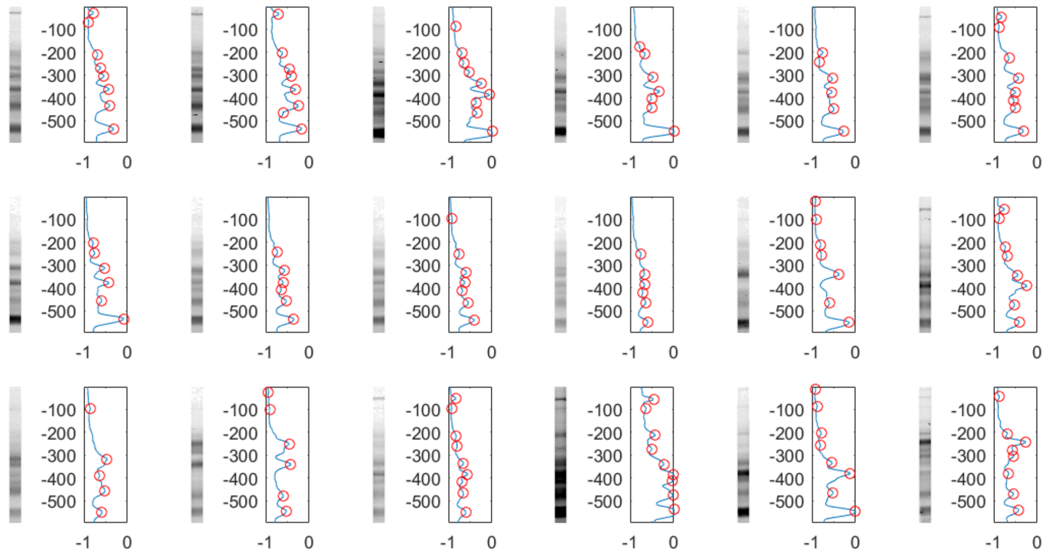

Supplementary Figure 1: Individual lanes and intensity graphs with detected peaks marked with a red circle. The software used and its code is under https://github.com/ha-ruiz75/BandDetection.git

Supplementary Figure S2

Multiple Sequence Alignment of Membrane Associated Proteins with Tryptophan (W) exchanges. The position of the alignment represents the region of the protein where Tryptophan and the alternative amino acid can be found after aligning 250 proteins sequences of H. pylori. Sequences aligned to the left side symbolize that the region of interest where tryptophan variations are present are at the N-terminal region of protein; aligned to the right represents it to be C-terminal, and centered alignments represent variations in the central region of the protein. For Color Coding information, see at the end of this figure. With the name of the protein locus tag it is described if the variation of Tryptophan takes place or not in the transmembrane region of the proteins, as well the predominant amino acid exchange in all aligned proteins.

HP_0099 (Transmembrane) Aa 13 (W::A::C)	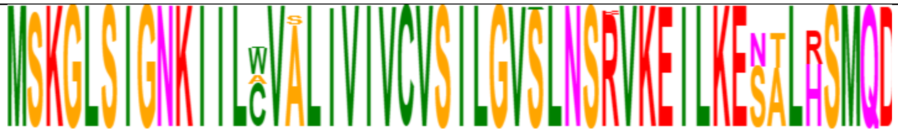
HP_0286 (No transmembrane) Aa 547 (W::L)	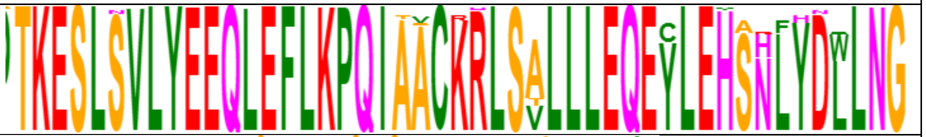
HP_0342 (Transmembrane) Aa 42 (W::S)	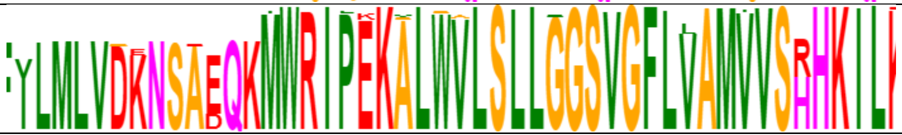
HP_0342 (Transmembrane) Aa 92 (W::G)	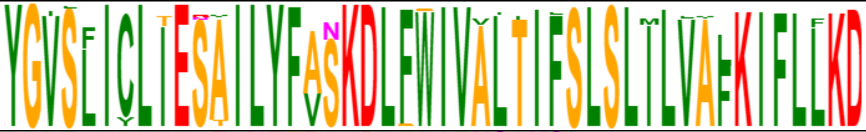
HP_0415 (No Transmembrane) Aa 3 (W::L);Aa 6(W::Y)	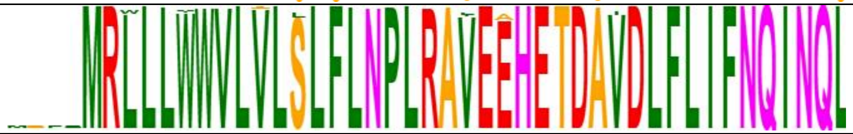
HP_0415 (No Transmembrane) Aa 269 (W::R)	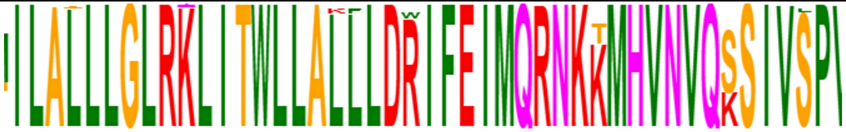
HP_0759 (No Transmembrane) Aa 201 (W::L)	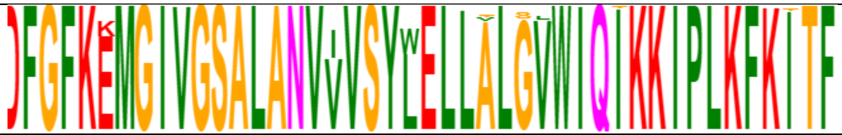

HP_1072 (No Transmembrane) Aa 201 (W::L)	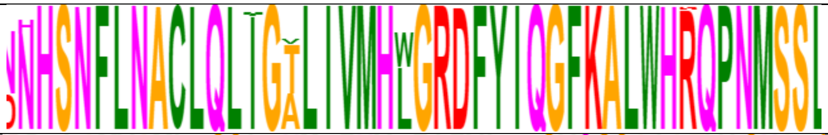
HP_1184 (Transmembrane) Aa 184 (W::L)	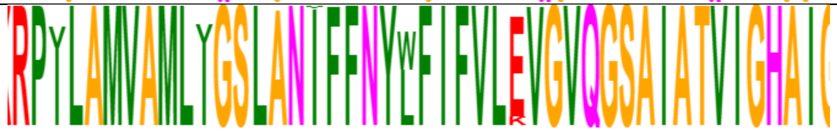
HP_1349 (No transmembrane) Aa 38 (W::L)	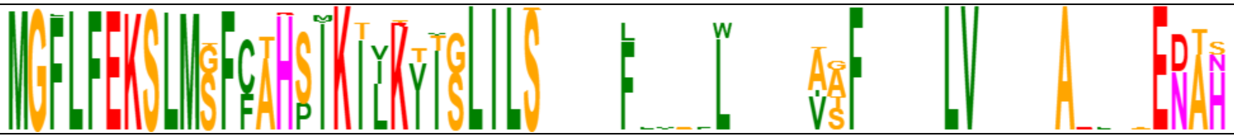
HP_1349 (No transmembrane) Aa 116 (W::L)	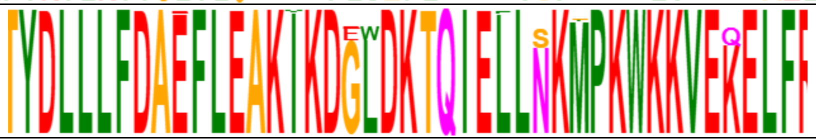

HP_0289 (HPP12_0288)

HP_0289	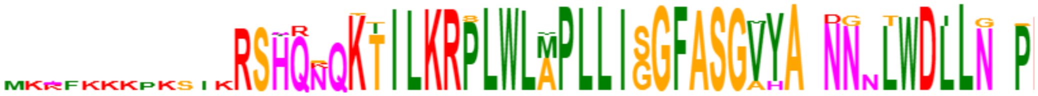
HP_0289	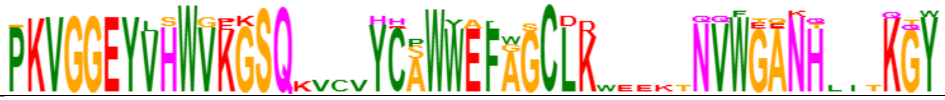
HP_0289	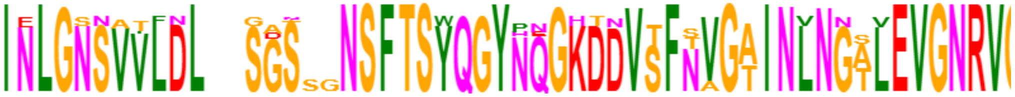
HP_0289	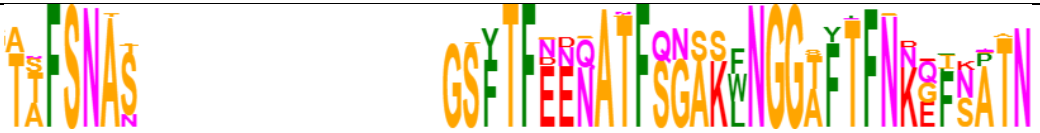

Modified Lesk Color scheme (described in http://www.bioinformatics.nl/~berndb/aacolour.html)

Small nonpolar	G, A, S, T	**Orange**
Hydrophobic	C, V, I, L, P, F, Y, M, W	**Green**
Polar	N, Q, H	**Magenta**
Charged	D, E, K, R	**Red**

Supplementary Table 1

	Genotypes			Phenotypes		
Strain	***cagA***	***vacA***	***hopQ***	**CagA**	**VacA**	**OipA**
049A33	+	s1/m1	Allele I	+	+	+
049A44	+	s1/m1	Allele I	+	+	+
098A14	-	s2/m2	Allele II	-	+	-
098C6	-	s2/m2	Allele II	-	-	-
101A27	+	s1/m1	Allele I	-	+	+
101A35	+	s1/m1	Allele I	+	+	+
104A27	-	s2/m2	Allele II	-	+	-
112A2	-	s2/m2	Allele II	-	-	-
112A5	-	s2/m2	Allele II	-	-	-
137C34	-	s2/m2	Allele I	-	-	-
139A39	-	s2/m2	Allele II	-	+	-
141C23	+	s1/m1	Allele II	+	+	+
145A30	-	s2/m2	Allele I	-	-	-
163A15	+	s1/m1	Allele II	+	+	+
163A4	-	s2/m2	Allele II	-	-	-

Supplementary Table 1: Strains used for analysis with cagA, vacA and hopQ genotypes, as well with CagA, VacA and OipA phenotypes determined by immunoblotting.

Supplementary Table 2

STRAIN	RAPD	TRYF	MLST	GOLD_STANDARD (MLST AND GENOTYPING)	SIMILAR TO…
**049A33**	0	0	1	0	49A44
**049A44**	0	0	1	0	49A33
**098A14**	1	1	0	1	98C6
**098C6**	1	1	0	1	98A14
**101A27**	0	1	1	1	
**101A35**	0	1	1	1	
**104A27**	1	1	1	1	
**112A2**	0	0	0	0	112A5
**112A5**	0	0	0	0	112A2
**139A39**	1	1	1	1	
**163A15**	1	1	1	1	
**163A4**	1	1	1	1	
**164A11**	0	0	0	1	164A4
**164A4**	0	0	0	1	164A11
**179A35**	1	1	1	1	
**203A5**	0	0	1	1	
**203C21**	0	1	1	1	
**203C9**	0	0	1	1	
**205A35**	1	1	1	1	
**205C19**	1	1	1	1	
**205C29**	1	1	1	1	
**211A23**	0	1	1	1	
**211A5**	0	1	1	1	
**219A10**	1	1	1	1	
**219C49**	1	1	1	1	
**230A1**	1	0	0	0	230A9
**230A13**	0	0	1	1	
**230A14**	0	1	1	1	
**230A9**	1	0	0	0	230A1
**232A3**	0	0	1	0	
**232A49**	1	0	1	0	
**243A22**	0	0	1	0	
**243A27**	0	0	1	0	
**272C12**	1	1	1	1	
**276C48**	1	1	1	1	
**281A17**	1	0	0	0	281C19
**302A4**	0	0	0	0	302A41
**302A41**	0	0	0	0	302A4
**318A44**	1	1	1	1	

**327C13**	1	1	0	0	171C4 and 171C18
**358A11**	1	1	1	1	
**374A3**	0	0	1	0	
**374A42**	0	0	1	0	
**374A5**	0	1	1	1	

Supplementary Table 2: 44 strains were tested for uniqueness using 4 different methods: RAPD, TryF, MLST and as Gold Standard was considered a combination of MLST and genotyping of strains for cagA, vacA (m1,m2 and s1,s2), hopQ (Allele 1 or 2), oipA and cagY. Number 0 describes strains as "not unique", while number 1 are those found to be "unique" by the respective method. When found to be similar to another strains, in any of the methods, the corresponding strain was annotated in column "similar to…".

Supplementary Table 3

		Gold Standard		
		Not Unique	Unique	Total
TryF	Not Unique	15	5	20
	Unique	1	23	24
Total		16	28	44
RAPD	Not Unique	11	12	23
	Unique	5	16	21
Total		16	28	44
MLST	Not Unique	8	4	12
	Unique	8	24	32
Total		16	28	44

Supplementary Table 3: Performance for TryF, RAPD and MLST techniques vs the Gold Standard (MLST and Genotyping of cagA, vacA, oipA, hopQ and cagY).

Supplementary Table 4

Primer	Sequence (5'-3')	Gene	Reference
CagTF	ACCCTAGTCGGTAATGGG	cagA	*Yamaoka et al*. [1]
CagTR	GCTTTAGCTTCTGATACC		
Va1-F	ATGGAAATACAACAAACACAC	vacA	Mukhopadhyay *et al.[2]*
Va1-R	CTGCTTGAATGCGCCAAAC		
Va3-F	GGTCAAAATGCGGTCATGG	vacA	Mukhopadhyay *et al.*
Va3-R	CCATTGGTACCTGTAGAAAC		
Va4-F	GGAGCCCCAGGAAACATT	vacA	Mukhopadhyay *et al.*
Va5-R	CATAACTAGCGCCTTGCAC		
OP5136	CAACGATAATGGCACAAACT	hopQ	Cao *et al*.[3]
OP4829	GTCGTATCAATAACAGAAGTTG		
BA8363	TCCAATCCAGAAGCGATTAA	hopQ	Cao *et al*.

Supplementary Table 4: Primers used for genotypification of strains.

Supplementary Table 5

Antibody	**Laboratory ID**	**Origin**	**Final concentration**	**Reference**
	Primary Antibodies			
CagA	AK268	Polyclonal antibody against N-terminal part of CagA (rabbit)	1:10000	*Hohlfeld et al.*[4]
α VacA	AK197	Polyclonal antibody against VacA (rabbit)	1:10000	Schmitt *et al.*[5]
α OipA	AK282	Polyclonal antibody against OipA (rabbit)	1:10000	Takahiko *et al.*[6]

Supplementary Table 5 Antibodies used for phenotyping of strains by immunoblotting

Supplementary Table 6

List of 51 proteins found to have a higher than expected changes in tryptophan content while comparing homologous proteins from H. pylori strains 26695 and P12. Locus tag used from strain 26695. Graphical representation of classes number of genes they include are shown in Figure 3B.

Locus_tag	Name	Classification	GO classes	UniProt link
**HP_0566**	Diaminopimelate epimerase DapF	Cytoplasm	GO:0009089,GO:0005737, GO:0008837	http://www.uniprot.org/uniprot/O25290
**HP_1046**	Ribosome maturation factor RimP		GO:0042274,GO:0005737	http://www.uniprot.org/uniprot/O25687
**HP_0099**	Methyl-accepting chemotaxis protein (TlpA)	Membrane associated	GO:0004871,GO:0016021	http://www.uniprot.org/uniprot/O24925
**HP_0286**	Cell division protein (FtsH)		GO:0051301,GO:0005524, GO:0016021,GO:0004222	http://www.uniprot.org/uniprot/O25060
**HP_0289**	Toxin-like outer membrane protein		GO:0042802	http://www.uniprot.org/uniprot/O25063
**HP_0342**	Uncharacterized protein		GO:0016021	http://www.uniprot.org/uniprot/O25109
**HP_0415**	Uncharacterized MscS family protein HP_0415		GO:0055085,GO:0016021, GO:0005886	http://www.uniprot.org/uniprot/O25170
**HP_0599**	Hemolysin secretion protein (HylB)		GO:0016020,GO:0004871	http://www.uniprot.org/uniprot/O25321
**HP_0743**	Peptidoglycan glycosyltransferase MrdB		GO:0005886,GO:0071555, GO:0008955,GO:0008360, GO:0009252,GO:0051301, GO:0007049,GO:0016021	http://www.uniprot.org/uniprot/P56098
**HP_0759**	Conserved hypothetical integral membrane protein		GO:0015238,GO:0016021, GO:0015297	http://www.uniprot.org/uniprot/O25454
**HP_0818**	Osmoprotection protein (ProWX)		GO:0006810,GO:0016021, GO:0005215,GO:0005886	http://www.uniprot.org/uniprot/O25496
**HP_1107**	Outer membrane protein (Omp23)			http://www.uniprot.org/uniprot/O25735

**HP_1184**	Conserved hypothetical integral membrane protein		GO:0015238,GO:0016021, GO:0015297	http://www.uniprot.org/uniprot/O25796
**HP_1349**	Uncharacterized protein		GO:0016021	http://www.uniprot.org/uniprot/O25904
**HP_1072**	Copper-transporting ATPase (CopA)	Membrane associated, cation binding	GO:0004008,GO:0005524, GO:0046872,GO:0016021, GO:0005886	http://www.uniprot.org/uniprot/P55989
**HP_0091**	Type II restriction Enzyme	Cation binding	GO:0009036,GO:0003677, GO:0009307	http://www.uniprot.org/uniprot/O24917
**HP_0142**	A/G-specific adenine glycosylase (MutY)		GO:0003824,GO:0006284, GO:0003677	http://www.uniprot.org/uniprot/O24954
**HP_0312**	Conserved hypothetical ATP-binding protein		GO:0005524	http://www.uniprot.org/uniprot/O25082
**HP_0656**	Cyclic dehypoxanthine futalosine synthase MqnC		GO:0005506,GO:0009234, GO:0046992,GO:0051539, GO:0016765	http://www.uniprot.org/uniprot/O25370
**HP_0661**	Ribonuclease HI RnhA		GO:0004523,GO:0046872, GO:0005737,GO:0003676	http://www.uniprot.org/uniprot/P56120
**HP_0793**	Peptide deformylase (Def)		GO:0006412,GO:0042586, GO:0046872	http://www.uniprot.org/uniprot/P56419
**HP_0860**	D-glycero-beta-D-manno-heptose-1,7-bisphosphate 7-phosphatase (GmhB)		GO:0034200,GO:0000287, GO:0005737,GO:0097171, GO:0009244,GO:0008270	http://www.uniprot.org/uniprot/O25531
**HP_1100**	Phosphogluconate dehydratase (Edd)		GO:0004456,GO:0019521, GO:0009255,GO:0046872, GO:0051539	http://www.uniprot.org/uniprot/P56111
**HP_1282**	Anthranilate synthase component 1 (TrpE)		GO:0000162,GO:0046872, GO:0004049	http://www.uniprot.org/uniprot/O25869
**HP_1213**	Polyribonucleotide nucleotidyltransferase (Pnp)	Metal-ion binding, Cytoplasm	GO:0006396,GO:0046872, GO:0004654,GO:0003723, GO:0005737,GO:0006402	http://www.uniprot.org/uniprot/O25812
**HP_0104**	2',3'-cyclic-nucleotide 2'-phosphodiesterase (CpdB)	Metal-ion binding, DNA binding	GO:0009166,GO:0016788, GO:0000166,GO:0046872	http://www.uniprot.org/uniprot/O24930

**HP_0043**	Mannose-6-phosphate isomerase (Pmi) or (AlgA)	Other	GO:0016779,GO:0000271, GO:0016853	http://www.uniprot.org/uniprot/O24884
**HP_0048**	Carbamoyltransferase HypF	Other	GO:0016743,GO:0016787, GO:0046944,GO:0003725, GO:0008270	http://www.uniprot.org/uniprot/O24889
**HP_0235**	Putative beta-lactamase HcpE		GO:0046677,GO:0005576, GO:0008800	http://www.uniprot.org/uniprot/O25021
**HP_0519**	Putative secreted protein			http://www.uniprot.org/uniprot/O25256
**HP_0558**	3-oxoacyl-[acyl-carrier-protein] synthase 2		GO:0006633,GO:0033817	http://www.uniprot.org/uniprot/O25284
**HP_0755**	Molybdopterin biosynthesis protein (MoeB)		GO:0008641	http://www.uniprot.org/uniprot/O25450
**HP_0965**	Uncharacterized protein		GO:0003924	
**HP_1105**	LPS biosynthesis protein		GO:0016757	http://www.uniprot.org/uniprot/O25733
**HP_1141**	Methionyl-tRNA formyltransferase (Fmt)		GO:0004479	http://www.uniprot.org/uniprot/P56461
**HP_1229**	Aspartokinase (LysC)		GO:0009088,GO:0009089, GO:0005524,GO:0019877, GO:0004072	http://www.uniprot.org/uniprot/O25827
**HP_1277**	Tryptophan synthase alpha chain (TrpA)		GO:0004834	http://www.uniprot.org/uniprot/P56141
**HP_1533**	Flavin-dependent thymidylate synthase (ThyX)		GO:0050660,GO:0006231, GO:0006235,GO:0050797	http://www.uniprot.org/uniprot/O26061
**HP_0036**	Putative protein	Unknown		
**HP_0064**	Uncharacterized protein			http://www.uniprot.org/uniprot/O24904
**HP_0108**	Uncharacterized protein			http://www.uniprot.org/uniprot/O24932
**HP_0270**	Uncharacterized protein			http://www.uniprot.org/uniprot/O25048
**HP_0430**	Uncharacterized protein			http://www.uniprot.org/uniprot/O25180
**HP_0568**	Uncharacterized protein			http://www.uniprot.org/uniprot/O25292

**HP_0744_2**	Partial ORF			
**HP_0762**	Uncharacterized protein			http://www.uniprot.org/uniprot/O25457
**HP_0783**	Uncharacterized protein	Unknown		http://www.uniprot.org/uniprot/O25472
**HP_0868**	Uncharacterized protein			http://www.uniprot.org/uniprot/O25538
**HP_0897**	Uncharacterized protein			http://www.uniprot.org/uniprot/O25557
**HP_0963**	Uncharacterized protein			http://www.uniprot.org/uniprot/O25616
**HP_1455**	Uncharacterized protein			http://www.uniprot.org/uniprot/O25994

Supplementary Images

The following are the original images from the Stainfree and RAPD analysis, used for Figure 1. Bacteria Gel 1 is the original image used for Figure 2A. Stainfree imaging software was set up to show overexposure through red staining of signals. In the case of RAPD, the metadata was not available (RAPD gel), while for Stainfree the metadata has been included (Gels 1-4)

Stainfree (Tryptophan imaging)

<table>
<tr><th>Gel image</th><th>Metadata</th></tr>
<tr><td>
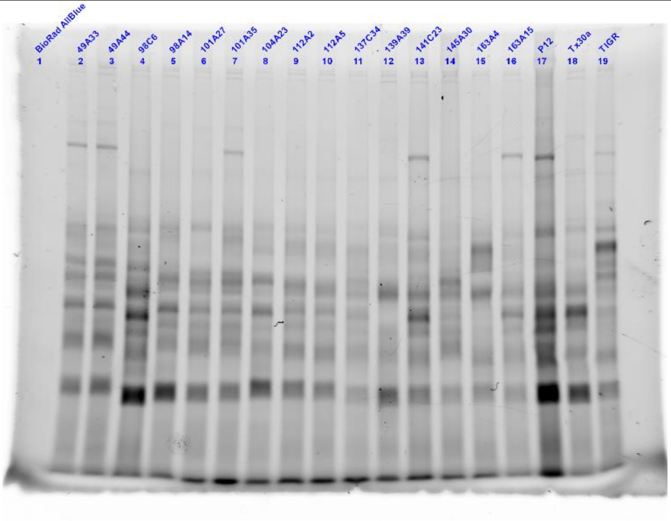

</td><td>
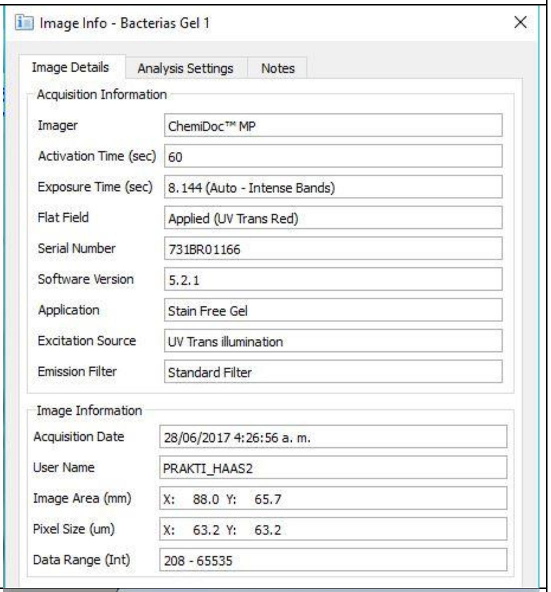
Image Info - Bacterias Gel 1
Image Details | Analysis Settings | Notes
Acquisition Information
Imager: ChemiDoc™ MP
Activation Time (sec): 60
Exposure Time (sec): 8.144 (Auto - Intense Bands)
Flat Field: Applied (UV Trans Red)
Serial Number: 731BR01166
Software Version: 5.2.1
Application: Stain Free Gel
Excitation Source: UV Trans illumination
Emission Filter: Standard Filter
Image Information
Acquisition Date: 28/06/2017 4:26:56 a. m.
User Name: PRAKTI_HAAS2
Image Area (mm): X: 88.0 Y: 65.7
Pixel Size (um): X: 63.2 Y: 63.2
Data Range (Int): 208 - 65535
</td></tr>
<tr><td>
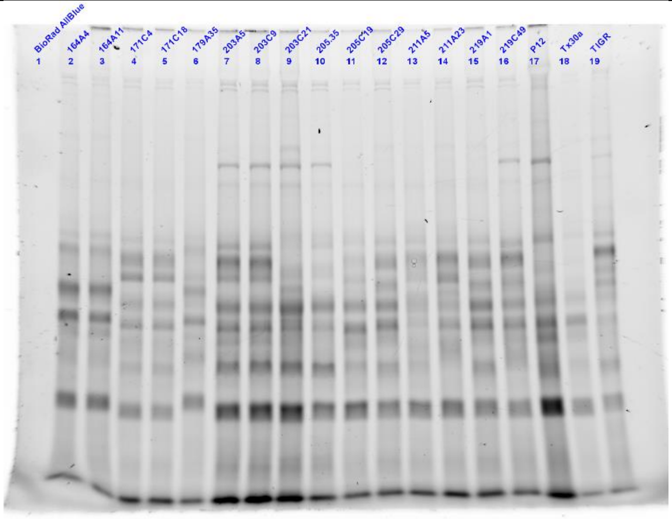

</td><td>
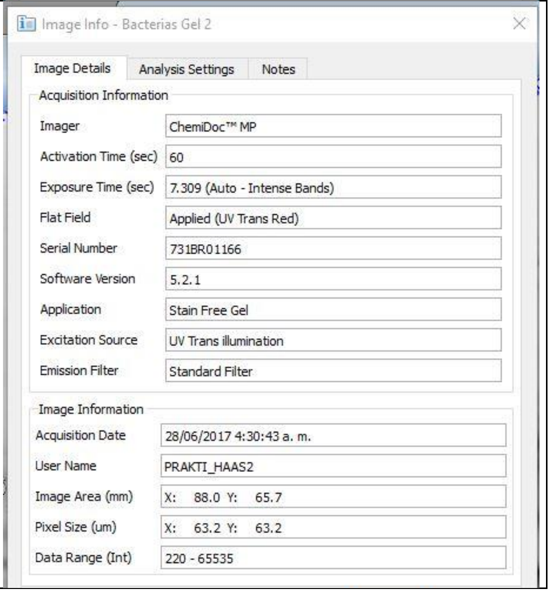
Image Info - Bacterias Gel 2
Image Details | Analysis Settings | Notes
Acquisition Information
Imager: ChemiDoc™ MP
Activation Time (sec): 60
Exposure Time (sec): 7.309 (Auto - Intense Bands)
Flat Field: Applied (UV Trans Red)
Serial Number: 731BR01166
Software Version: 5.2.1
Application: Stain Free Gel
Excitation Source: UV Trans illumination
Emission Filter: Standard Filter
Image Information
Acquisition Date: 28/06/2017 4:30:43 a. m.
User Name: PRAKTI_HAAS2
Image Area (mm): X: 88.0 Y: 65.7
Pixel Size (um): X: 63.2 Y: 63.2
Data Range (Int): 220 - 65535
</td></tr>
</table>

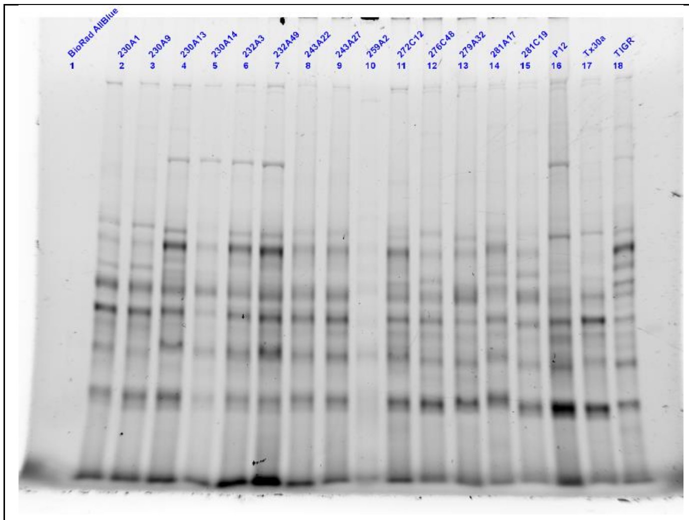

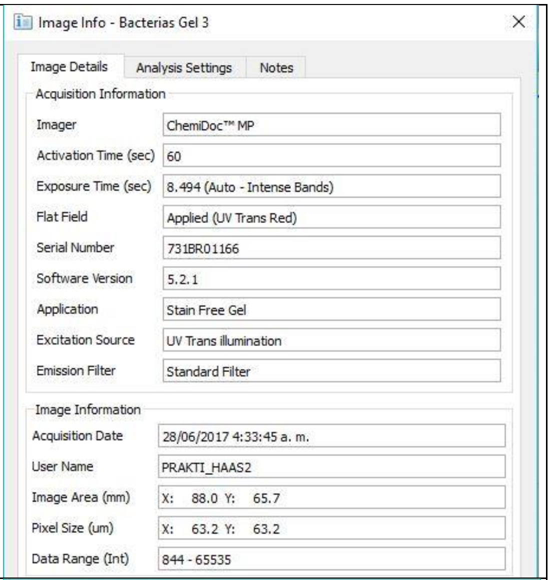

Image Info - Bacterias Gel 3

Image Details | Analysis Settings | Notes

Acquisition Information	
Imager	ChemiDoc™ MP
Activation Time (sec)	60
Exposure Time (sec)	8.494 (Auto - Intense Bands)
Flat Field	Applied (UV Trans Red)
Serial Number	731BR01166
Software Version	5.2.1
Application	Stain Free Gel
Excitation Source	UV Trans illumination
Emission Filter	Standard Filter

Image Information	
Acquisition Date	28/06/2017 4:33:45 a. m.
User Name	PRAKTI_HAAS2
Image Area (mm)	X: 88.0 Y: 65.7
Pixel Size (um)	X: 63.2 Y: 63.2
Data Range (Int)	844 - 65535

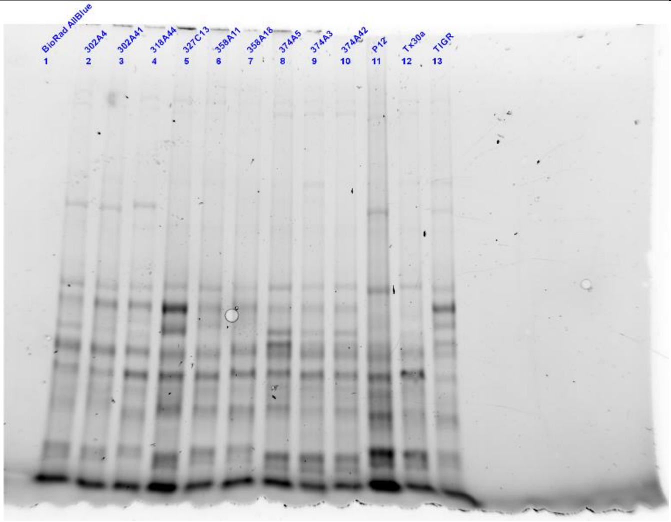

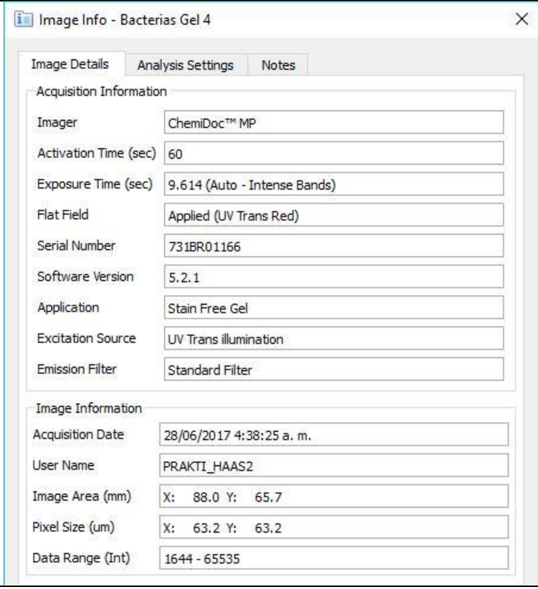

Image Info - Bacterias Gel 4

Image Details | Analysis Settings | Notes

Acquisition Information	
Imager	ChemiDoc™ MP
Activation Time (sec)	60
Exposure Time (sec)	9.614 (Auto - Intense Bands)
Flat Field	Applied (UV Trans Red)
Serial Number	731BR01166
Software Version	5.2.1
Application	Stain Free Gel
Excitation Source	UV Trans illumination
Emission Filter	Standard Filter

Image Information	
Acquisition Date	28/06/2017 4:38:25 a. m.
User Name	PRAKTI_HAAS2
Image Area (mm)	X: 88.0 Y: 65.7
Pixel Size (um)	X: 63.2 Y: 63.2
Data Range (Int)	1644 - 65535

RAPD images

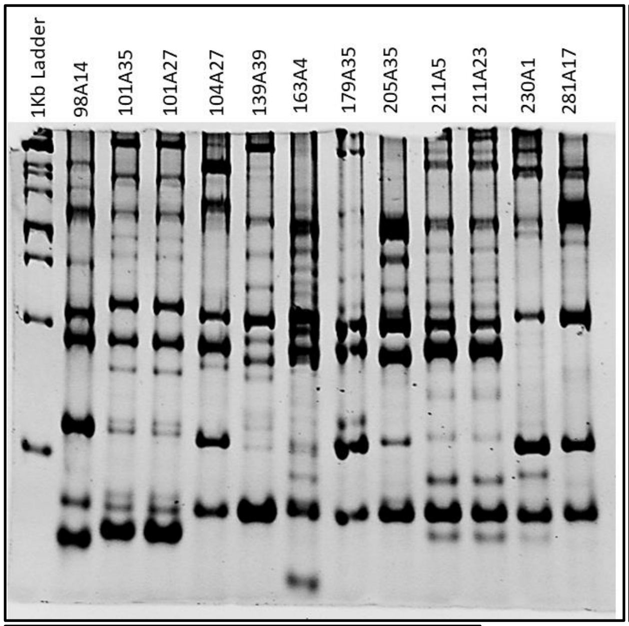
1Kb Ladder
98A14
101A35
101A27
104A27
139A39
163A4
179A35
205A35
211A5
211A23
230A1
281A17

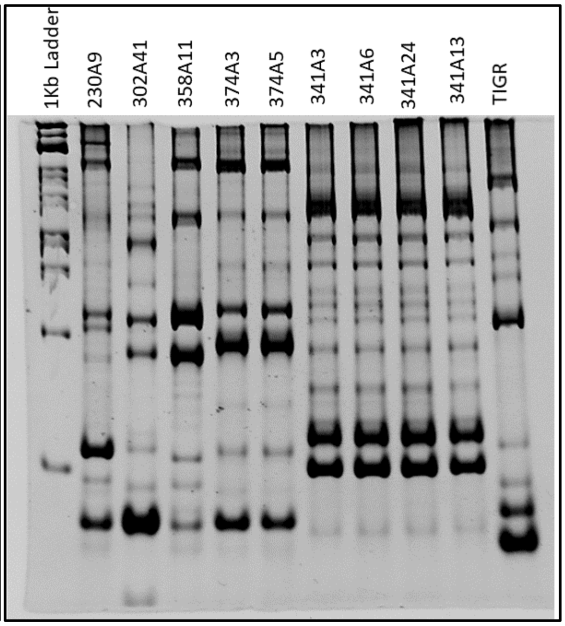
1Kb Ladder
230A9
302A41
358A11
374A3
374A5
341A3
341A6
341A24
341A13
TIGR

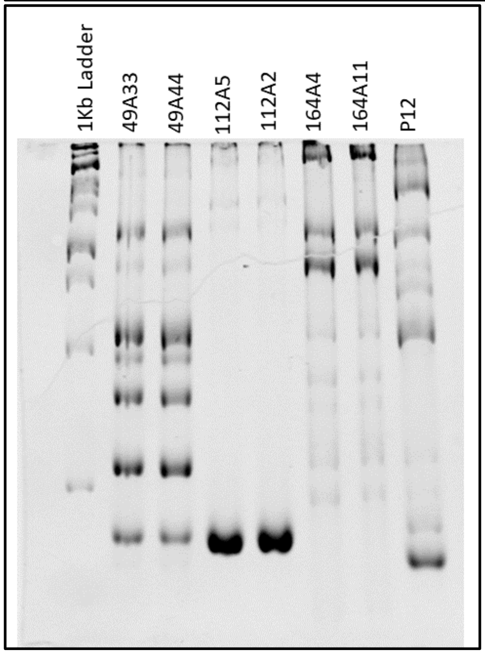
1Kb Ladder
49A33
49A44
112A5
112A2
164A4
164A11
P12

References Supplementary Section

[1] Y. Yamaoka, M. S. Osato, A. R. Sepulveda, O. Gutierrez, N. Figura, J. G. Kim, T. Kodama, K. Kashima, and D. Y. Graham, "Molecular epidemiology of Helicobacter pylori: separation of H. pylori from East Asian and non-Asian countries," *Epidemiology and Infection,* vol. 124, pp. 91-96, 2000.

[2] A. K. Mukhopadhyay, D. Kersulyte, J.-Y. Jeong, S. Datta, Y. Ito, A. Chowdhury, S. Chowdhury, A. Santra, S. K. Bhattacharya, T. Azuma, G. B. Nair, and D. E. Berg, "Distinctiveness of Genotypes of Helicobacter pylori in Calcutta, India," *J Bacteriol,* vol. 182, pp. 3219-3227, June 1, 2000 2000.

[3] P. Cao and T. L. Cover, "Two different families of hopQ alleles in Helicobacter pylori," *J Clin Microbiol,* vol. 40, pp. 4504-11, Dec 2002.

[4] S. Hohlfeld, I. Pattis, J. Puls, G. V. Plano, R. Haas, and W. Fischer, "A C-terminal translocation signal is necessary, but not sufficient for type IV secretion of the Helicobacter pylori CagA protein," *Mol Microbiol,* vol. 59, pp. 1624-37, Mar 2006.

[5] W. Schmitt and R. Haas, "Genetic analysis of the Helicobacter pylori vacuolating cytotoxin: structural similarities with the IgA protease type of exported protein," *Mol Microbiol,* vol. 12, pp. 307-19, Apr 1994.

[6] T. Kudo, Z. Z. Nurgalieva, M. E. Conner, S. Crawford, S. Odenbreit, R. Haas, D. Y. Graham, and Y. Yamaoka, "Correlation between Helicobacter pylori OipA Protein Expression and oipA Gene Switch Status," *Journal of Clinical Microbiology,* vol. 42, pp. 2279-2281, May 1, 2004 2004.
